# Dataset of urban development analysis in a section of Kuje Area Council, Abuja, Nigeria

**DOI:** 10.1016/j.dib.2022.108777

**Published:** 2022-11-23

**Authors:** Chima Iheaturu, Chukwuma Okolie, Emmanuel Ayodele, Andy Egogo-Stanley, Solomon Musa, Chinwe Ifejika Speranza

**Affiliations:** a University of Bern, Institute of Geography, Bern, Switzerland; b University of Lagos, Department of Surveying and Geoinformatics, Lagos, Nigeria; c Federal Capital Development Authority, Department of Survey and Mapping, Abuja, Nigeria

**Keywords:** Change detection analysis, Digital map creation, Geospatial methods, Google earth historical imagery, Parsimonious land use, SfM photogrammetry

## Abstract

Urban development will likely continue to increase in suburban areas to cater for the growing human population. In Nigeria, the relevant analysis of these urban developments is not well documented. This article presents spatiotemporal datasets for analysing urban developments in a suburb of Kuje, an Area Council within the Federal Capital Territory of Nigeria. Data from Google Earth (GE) historical imagery of 2005 was used as a baseline for analysis and was compared with a UAV digital orthomosaic of 2019 to quantify urban developments. This data provides useful information on the status of urban development that has taken place in the Kuje suburb over 14 years. The data will be of great importance to town planners and urban development authorities for future planning, and for making informed decisions about urban development issues in the area.

**Specifications Table**

**Table:** 

Subject	Environmental science
Specific subject area	Urban development analysis
Type of data	Image Table Figure Shapefile
How the data were acquired	Google Earth (GE) imagery: Google Earth Pro GE imagery extraction software: Elshayal Smart GIS Unmanned Aerial Vehicle (UAV): DJI Mavic 2 Pro Flight planning software: DroneDeploy UAV data processing software: Pix4D Mapper Ground control points (GCPs) measurement instrument: Hi-target V30 RTK Global Positioning System (GPS) GPS data recording software: Hi-Survey Road Mapping software: ArcMap 10.8
Data format	Raw Analysed
Description of data collection	The 2005 high-resolution GE imageries of the study area were extracted at optimal zoom level using the Elshayal Smart GIS software. Also, DJI Mavic 2 Pro was used to acquire the images of the study area in 2019. The UAV was flown at a low flying height (61 m) to achieve a high resolution. Hi-target RTK was used to determine the positions of the GCPs while the UAV images were processed to generate an orthomosaic.
Data source location	City/Town/Region: Kuje, Abuja, Federal Capital Territory Country: Nigeria Latitude and longitude (and GPS coordinates, if possible) for collected samples/data: Latitude 7° 14’ 7” N to 7° 14’ 28” N and Longitude 8° 52’ 37” E to 8° 52’ 54” E
Data accessibility	Repository name: Mendeley Data Data identification number: 10.17632/63n37 × 7c92.1 Direct URL to data: https://data.mendeley.com/datasets/63n37x7c92
Related research article	Iheaturu, C., Okolie, C., Ayodele, E., Egogo-Stanley, A., Musa, S., Speranza, C.I., 2022. A simplified structure-from-motion photogrammetry approach for urban development analysis. Remote Sens Appl 100850. https://doi.org/10.1016/J.RSASE.2022.100850

## Value of the Data

• The datasets provide useful information on the status of urban development that has taken place in the Kuje suburb between 2005 and 2019. The UAV data can be used as a baseline for future monitoring of the urban changes and developments in the area.
• The data can benefit researchers and town planners interested in experimental data and urban development analysis.
• Town planners, as well as urban development authorities, can use the data to make informed decisions on urban development issues in the area. In addition, the data can aid policy formulation to achieve parsimonious use of land in the area.

## 1 Objective

Rapid and uncontrolled urban development in Nigerian cities and suburbs has made urban development analysis very important for urban planning and policy-making. Iheaturu et al., [2] presented a simplified method for conducting urban development analysis. This method combined readily available Google Earth® historical imagery (baseline data) and UAV-derived imagery (recent data) to analyse urban development. The paper aimed to outline a practical workflow that can be easily adopted by urban planners and researchers alike. Although the method was presented in sufficient detail to allow for its replicability, the goal of the paper cannot be fully achieved unless the data is also made available. Thus, the key objective of this data article is to provide a well-described dataset of the urban development analysis performed by Iheaturu et al., [2] both in raw and analysed formats to allow urban planners and researchers alike to reproduce or reuse them. The publication of this dataset and associated metadata, including the release into the public domain advances the goals of the FAIR Guiding Principles for scientific data management and stewardship.

## 2 Data Description

**Fig. 1:**
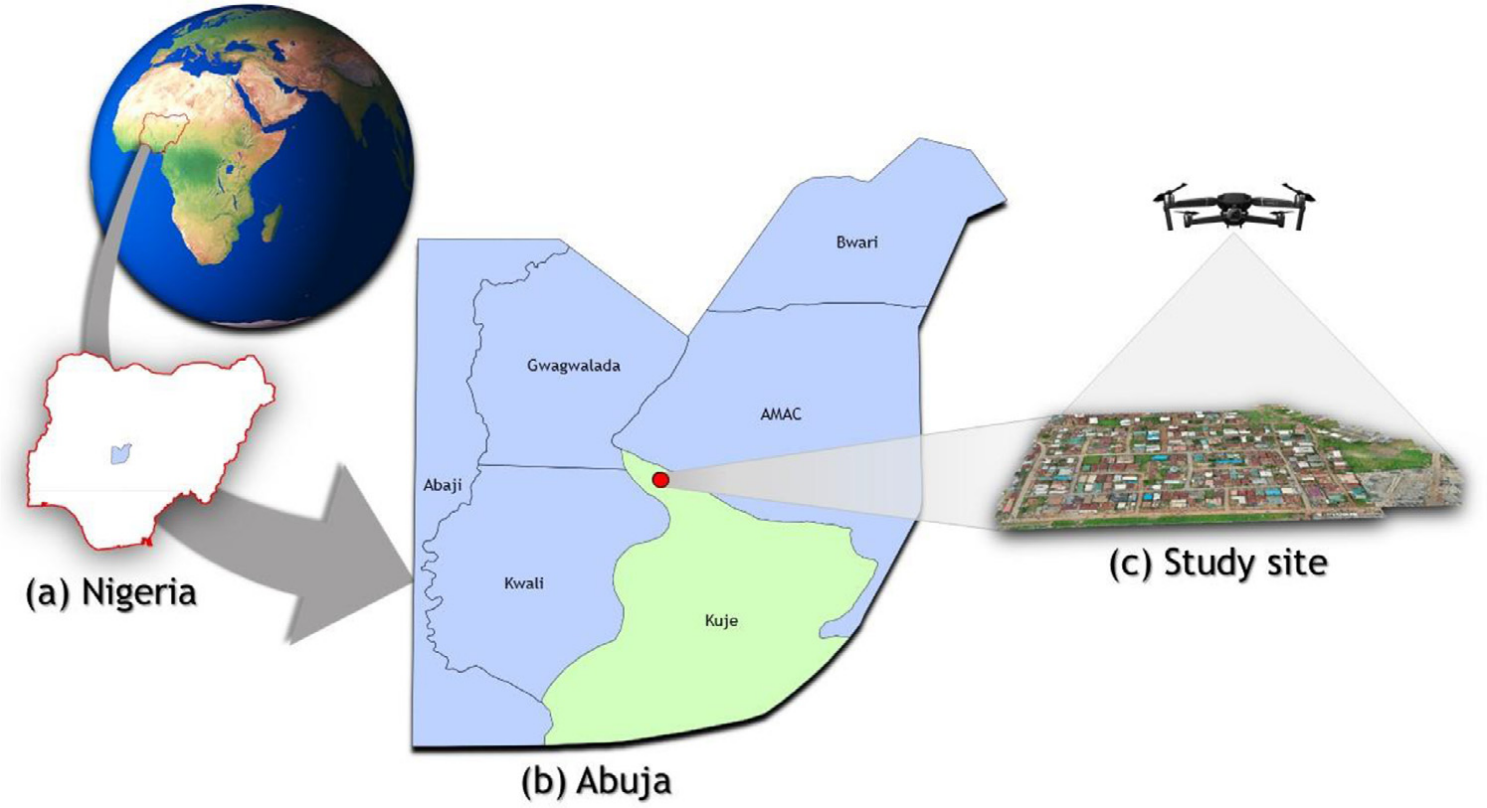
Study area: (a) Nigeria's location on the globe and an outline of Nigeria's administrative map showing the location of Abuja (highlighted in blue), (b) the map of Abuja showing the location of Kuje Area Council (highlighted in green), as well as the location of the study site, indicated using a red dot, and (c) visualisation of the DJI Mavic 2 Pro capturing the images of the study site.

In this section, a description of the datasets used for urban development analysis using GE historical imageries and UAV data is presented. The data description covers a small section of Kuje in Abuja as shown in Fig. 1. (folder dataset “Figure” https://data.mendeley.com/datasets/63n37x7c92). The dataset contains a figure, Google Earth Historical data, UAV and GCPs data, and the results of the Urban development analysis conducted.

### 2.1 Google Earth historical image data

Google Earth's “Historical Imagery” enables users to view images and observe changes over a region at different epochs [4]. Usually, the criteria for selecting a particular year include the degree of cloud cover and image clarity. Google Earth images are spatially referenced to the WGS84 datum, which can be considered equivalent to the GRS80 ellipsoid, for practical purposes [1]. Although there is little documentation about the metadata of Google Earth images [5], they have found wide uses in applications such as land use change detection, modelling of earth surface processes, habitat availability, health and surveillance systems, agriculture, landscape and other applications etc. [3], [4], [5]. The 2005 GE Historical Imagery dataset consists of 42 images extracted from Google Earth Pro using Elshayal Smart GIS software (folder dataset “GE Historical Image data\Raw” https://data.mendeley.com/datasets/63n37x7c92). The images are in JPEG format with a dimension of 1299 × 728 pixels, and a resolution of 96 dots per inch (dpi), and each image comes with an associated world file (*jgw). These images were orthorectified and mosaicked into a composite (folder dataset “GE Historical Image data\Mosaicked” https://data.mendeley.com/datasets/63n37x7c92). The mosaicked data is saved as a Tagged Image File Format (*TIFF) file with its associated world file (*tfw).

### 2.2 UAV and GCPs data

A DJI Mavic 2 Pro UAV was used to capture the images of a small section of Kuje. The UAV is equipped with an in-built Global Navigation Satellite System (GPS and GLONASS) that enables the geolocation of acquired images. Nine GCPs were distributed in the area of interest and their coordinates were then measured using a Hi-target V30 GNSS unit in Real-Time Kinematic (RTK) mode. The UAV and GCPs data consist of 470 geolocated images (folder dataset “UAV and GCP data\UAV images\Raw images” https://data.mendeley.com/datasets/63n37x7c92) acquired using the DJI Mavic 2 Pro UAV. Each image was saved in JPEG format with a dimension of 5472 × 3648 pixels and a resolution of 72 dpi. The images were processed using Pix4D Mapper software to generate densified point cloud data (folder dataset “UAV and GCP data\Point cloud” https://data.mendeley.com/datasets/63n37x7c92). The point cloud data was saved in LiDAR file format (*.las) and contains 4,856,037 3D points. Nine GCPs (folder dataset “UAV and GCP data\GCP data” https://data.mendeley.com/datasets/63n37x7c92) acquired using the Hi-target V30 RTK GPS were used to accurately scale and georeference the point cloud data. The GCPs data is a Microsoft Excel table containing the GCPs coordinates. The point cloud was further processed to obtain an orthomosaic (folder dataset “UAV and GCP data\UAV images\Orthomosaic” https://data.mendeley.com/datasets/63n37x7c92). Subsequently, the visible urban features contained in both the “GE_images 2005_mosaicked” and the “Orthomosaic” were digitised using ArcMap 10.8 software to obtain the shapefiles for 2005 (folder dataset “GE Historical Images data\Shapefiles\2005” https://data.mendeley.com/datasets/63n37x7c92) and 2019 (folder dataset “UAV and GCP data\Shapefiles\2019” https://data.mendeley.com/datasets/63n37x7c92) respectively.

### 2.3 Urban development analysis results

Urban development analysis was conducted to ascertain the level of development that has taken place (folder dataset “Urban development statistics” https://data.mendeley.com/datasets/63n37x7c92). The urban development statistics are presented in a Microsoft Excel table with charts showing the urban developments in the area for the period (2005 – 2019).

## 3 Experimental Design, Materials and Methods

### 3.1 Overview

To analyse the urban developments, GE historical imagery of 2005 was employed to serve as the baseline data which was compared with UAV-acquired data of 2019. In addition, the heights of the buildings in the study site were evaluated to ascertain if the land has been used parsimoniously.

### 3.2 Google Earth historical imagery acquisition

Elshayal Smart GIS software was used to extract the GE historical images of 2005 from Google Earth Pro at optimal zoom level to get high-resolution images. By default, GE displays the most recent imagery within its archive. Hence, the time slider tool within GE Pro was used to fix the year to 2005. Next, the extracted individual images were orthorectified and then mosaicked into a composite image in ArcMap 10.8 software.

### 3.3 UAV and GCPs data acquisition

The UAV data was acquired using a DJI Mavic 2 Pro UAV on the 26th of July 2019 between 12:00 and 13:00 hours (GMT +1) local time to get the best illumination. However, before the UAV survey, nine GCP targets were evenly distributed around the study site and subsequently measured instantaneously using the Hi-target V30 RTK GPS instrument in high precision base and rover real-time kinematic (RTK) mode [6]. This arrangement uses a “differential GPS” system that works by having a known base station that measures the errors in the GPS signals and sends corrections to the rover [6]. These corrected GCP coordinates were then recorded using the Hi-Survey Road Software (installed on the Hi-Target controller).

The UAV flight planning was done using DroneDeploy software. Here, the flight height (61.3 m), flight path (single strip), horizontal velocity (5 m/s), forward (60%) and side (75%) overlap were set. The DJI Mavic 2 Pro UAV followed the predefined path capturing 470 nadir images at regular intervals. All the captured images were geolocated using the EXIF tags. The flight conditions during the UAV survey were clear sky with a wind velocity of less than 2 m/s. The geolocated images were imported into Pix4D Mapper and then processed using the parameters in Table 1 to generate the point cloud data, and orthomosaic.

**Table 1:** Parameters used in the Pix4D processing

Processing stages	Processing options		Settings
Initial Processing	Keypoint image scale		Quarter image size
	Matching image pairs		Aerial grid or corridor
	Targeted number of keypoints		Automatic

Point Cloud and Mesh	Point Cloud Densification	Image scale	Quarter image size, fast
		Point density	Optimal
		Minimum number of matches	3
		3D textured mesh setting	Medium resolution
		Matching window size	9 × 9 pixels

DSM, Orthomosaic and Index	DSM and Orthomosaic Resolution		Automatic
	DSM filters		Check “use noise filtering”
			Check “use surface smoothing”
	Raster DSM		GeoTIFF
	Orthomosaic		GeoTIFF

### 3.4 Urban development analysis

The GE Historical Imagery for 2005 as well as the orthomosaic for 2019 were visualised in ArcMap 10.8 software. Subsequently, the buildings, fences, and roads were digitised and saved as shapefiles. These features were regarded to be indicators of urban development. Thus, the urban development analysis carried out entailed comparing the shapefiles for both 2005 and 2019 to ascertain the level of development that occurred in the area.

## Ethics Statement

The authors declare that all ethical practices have been followed in relation to the development, writing, and publication of this article.

## CRediT Author Statement

**Chima Iheaturu:** Conceptualisation, Formal analysis, Methodology, Data curation, Visualisation, Writing – original draft, Writing – review & editing; **Chukwuma Okolie:** Conceptualisation, Formal analysis, Methodology, Writing – review & editing; **Emmanuel Ayodele:** Supervision, Writing – review & editing; **Andy Egogo-Stanley:** Methodology, Writing – review & editing; **Solomon Musa:** Methodology, Writing – review & editing; **Chinwe Ifejika Speranza:** Supervision, Writing – review & editing.

## Declaration of Competing Interest

The authors declare that they have no known competing financial interests or personal relationships that could have appeared to influence the work reported in this paper.

## Data Availability

Dataset of urban development analysis in a section of Kuje Area Council, Abuja (Original data) (Mendeley Data).
